# Phylogenetic reduction of the magnocellular red nucleus in primates and inter-subject variability in humans

**DOI:** 10.3389/fnana.2024.1331305

**Published:** 2024-03-13

**Authors:** Martin Stacho, A. Niklas Häusler, Andrea Brandstetter, Francesca Iannilli, Hartmut Mohlberg, Christian Schiffer, Jeroen B. Smaers, Katrin Amunts

**Affiliations:** ^1^C. and O. Vogt Institute for Brain Research, Medical Faculty and University Hospital Düsseldorf, Heinrich Heine University Düsseldorf, Düsseldorf, Germany; ^2^Institute of Neuroscience and Medicine, INM-1, Research Centre Jülich, Jülich, Germany; ^3^Department of Anthropology, Stony Brook University, Stony Brook, NY, United States

**Keywords:** red nucleus, human brain, primate brain, cytoarchitectonic probability maps, Julich-Brain, BigBrain, evolution

## Abstract

**Introduction:**

The red nucleus is part of the motor system controlling limb movements. While this seems to be a function common in many vertebrates, its organization and circuitry have undergone massive changes during evolution. In primates, it is sub-divided into the magnocellular and parvocellular parts that give rise to rubrospinal and rubro-olivary connection, respectively. These two subdivisions are subject to striking variation within the primates and the size of the magnocellular part is markedly reduced in bipedal primates including humans. The parvocellular part is part of the olivo-cerebellar circuitry that is prominent in humans. Despite the well-described differences between species in the literature, systematic comparative studies of the red nucleus remain rare.

**Methods:**

We therefore mapped the red nucleus in cytoarchitectonic sections of 20 primate species belonging to 5 primate groups including prosimians, new world monkeys, old world monkeys, non-human apes and humans. We used Ornstein-Uhlenbeck modelling, ancestral state estimation and phylogenetic analysis of covariance to scrutinize the phylogenetic relations of the red nucleus volume.

**Results:**

We created openly available high-resolution cytoarchitectonic delineations of the human red nucleus in the microscopic BigBrain model and human probabilistic maps that capture inter-subject variations in quantitative terms. Further, we compared the volume of the nucleus across primates and showed that the parvocellular subdivision scaled proportionally to the brain volume across the groups while the magnocellular part deviated significantly from the scaling in humans and non-human apes. These two groups showed the lowest size of the magnocellular red nucleus relative to the whole brain volume and the largest relative difference between the parvocellular and magnocellular subdivision.

**Discussion:**

That is, the red nucleus has transformed from a magnocellular-dominated to a parvocellular-dominated station. It is reasonable to assume that these changes are intertwined with evolutionary developments in other brain regions, in particular the motor system. We speculate that the interspecies variations might partly reflect the differences in hand dexterity but also the tentative involvement of the red nucleus in sensory and cognitive functions.

## Introduction

**Figure 1:**
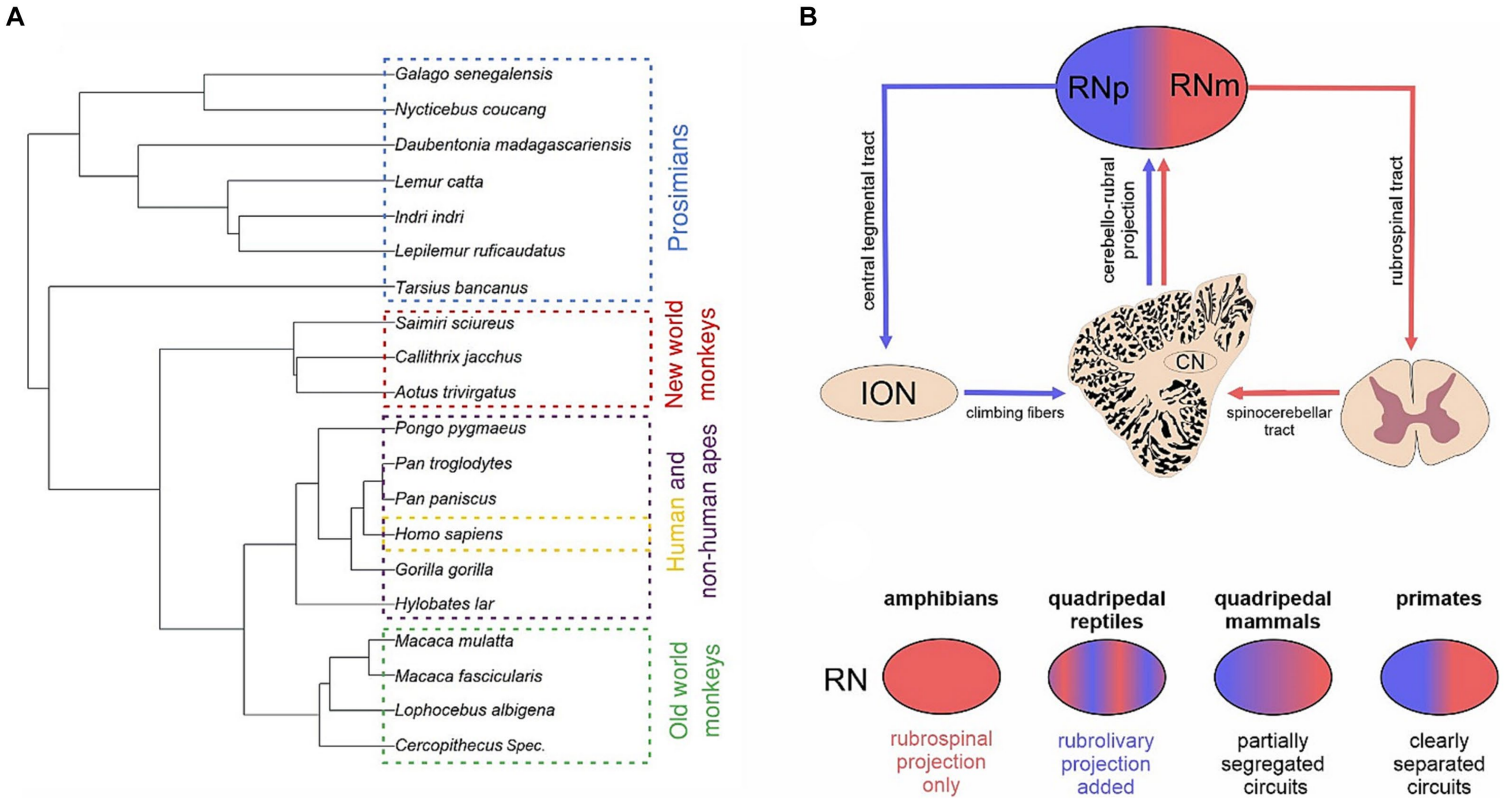
Phylogenetic considerations of the red nucleus. **(A)** Phylogenetic tree showing the phylogenetic relations between the species studied in this study. The species were divided into 5 groups, i.e., prosimians (blue), new world monkeys (red), old world monkeys (green), non-human apes (purple) and humans (yellow). **(B)** Schematic drawing showing the different main descending connections of the parvocellular (RNp) and magnocellular (RNm) red nucleus. While RNm gives rise to the rubrospinal tract, RNp projects to the inferior olivary nucleus (ION). Both subdivisions receive input from cerebellum via the cerebellar nuclei (CN). **(C)** Schematic illustration of major changes of the RN during the phylogeny as reviewed in Basile et al. (2021). While the amphibian RN projects to the spinal cord, the RN in quadrupedal reptiles also projects to the ION. The two projection neurons form partially segregated populations within the mammalian RN dividing it into the RNp and RNm, although such correspondence between the cytoarchitecture and connectivity might not apply to cats (see Pong et al., 2002). This distinction of the projection systems into the RNp and RNm is especially clear in primates.

The red nucleus (RN) is a large subcortical nucleus located in the ventromedial mesencephalon, ventraly to the centromedian thalamic nucleus, dorsomedialy to the substantia nigra, and ventromedialy to the subthalamic nucleus. The RN is subdivided into the caudal, magnocellular red nucleus (RNm) and the rostral, parvocellular red nucleus (RNp). The former gives rise to the rubrospinal tract, while RNp projects to the inferior olivary nucleus via the central tegmental tract and is part of the cerebro-rubro-olivo-cerebellar loop (Ralston, 1994b; Azizi, 2007; Onodera and Hicks, 2009; Habas et al., 2010; Novello et al., 2022; Figure 1B). However, there are striking differences with respect to the RN, its organization and circuitry across vertebrates (ten Donkelaar, 1988; Miller and Gibson, 2009; Gruber and Gould, 2010 for recent reviews see Basile et al., 2021; Olivares-Moreno et al., 2021; Figure 1C).

The emergence of RN in the phylogeny seems to be associated with the occurrence of limb like structures (incl. pectoral fins) used for locomotion (ten Donkelaar, 1988; Gruber and Gould, 2010; Olivares-Moreno et al., 2021). With certain exceptions, some form of RN and/or the rubrospinal tract have been identified in finned fishes incl. cartilaginous as well as bony fishes (Smeets and Timerick, 1981; Ronan and Northcutt, 1985; New et al., 1998; Nakayama et al., 2016; Yamamoto et al., 2017) but not in jawless fishes devoid of fins such as the lamprey and hagfish (Ronan, 1989; Pombal and Megías, 2020). The RN might have played a crucial role in the evolution of land locomotion in terrestrial vertebrates. While in amphibians an ill-defined RN is part of a primitive relay circuit between the cerebellum and spinal cord, the rubro-olivary and olivo-cerebellar projection is added to the circuit in quadrupedal reptiles (for a review see Basile et al., 2021; Figure 1C). Furthermore, in quadrupedal mammals these rubrospinal and rubro-olivo-cerebellar components develop into partially segregated networks with distinct cerebellar nuclei involved in each of the two circuits (but see Pong et al., 2002). Especially in rodents (e.g., in rats, mice, and rabbits) and carnivores (e.g., in cats) a gradual subdifferentiation of RN into RNp and RNm is apparent and a much more clear-cut distinction appears in primates (Burman et al., 2000a,b; Basile et al., 2021; Figure 1C).

In macaques, the more caudally located RNm contains large rubrospinal neurons with variable morphologies and long spiny dendritic ramifications (King et al., 1971; Burman et al., 2000a). On the other hand, the more rostrally located RNp contains rubro-olivary projection neurons that are smaller than the rubrospinal neurons in RNm. The rubro-olivary neurons possess extensive, though less complex, dendritic ramifications with small dendritic spines. In addition, both RN subdivisions contain small local interneurons although they might be sparse in RNm (King et al., 1971; Burman et al., 2000a). Similar large, medium and small sized neurons have also been reported in the RN of baboons, gibbons and humans (Padel et al., 1981; Onodera and Hicks, 2009, 2010).

Early anatomical studies indicate that the rubrospinal tract in humans is rudimentary and from the few rubrospinal fibers only a fraction projects beyond upper cervical segments of the spinal cord (Massion, 1967; Nathan and Smith, 1982). The rubrospinal tract might have been reduced especially in bipedal primates, as the pyramidal tract might have successively dominated the locomotor function (Padel et al., 1981; Massion, 1988; Onodera and Hicks, 1999, 2009; Gruber and Gould, 2010; Herculano-Houzel et al., 2016). In humans, the rubrospinal tract is thus thought to only contribute to the locomotion of upper limbs with little or no influence over lower extremities (Nathan and Smith, 1982; Onodera and Hicks, 2009; Gruber and Gould, 2010). Given the sparseness of the rubrospinal tract in humans, the corresponding RNm, where the rubrospinal tract is supposed to originate, is small in size. Some even reported an absence of so called „giant “neurons in human RN that are typical for RNm in other mammals such as rats and monkeys (King et al., 1971; Patt et al., 1994). Nevertheless, the RNm can be delineated in the caudal RN sections in humans based on the cytoarchitecture (incl. large/giant neurons) (Onodera and Hicks, 2010) and calcium-binding proteins (Ulfig and Chan, 2001). Furthermore, by means of DTI-tractography, a putative RNm can be segmented within the RN based on its connection to the interposed cerebellar nucleus (Cacciola et al., 2019). Thus, the RNm does exist in humans but its functional relevance remains elusive.

**Figure 2:**
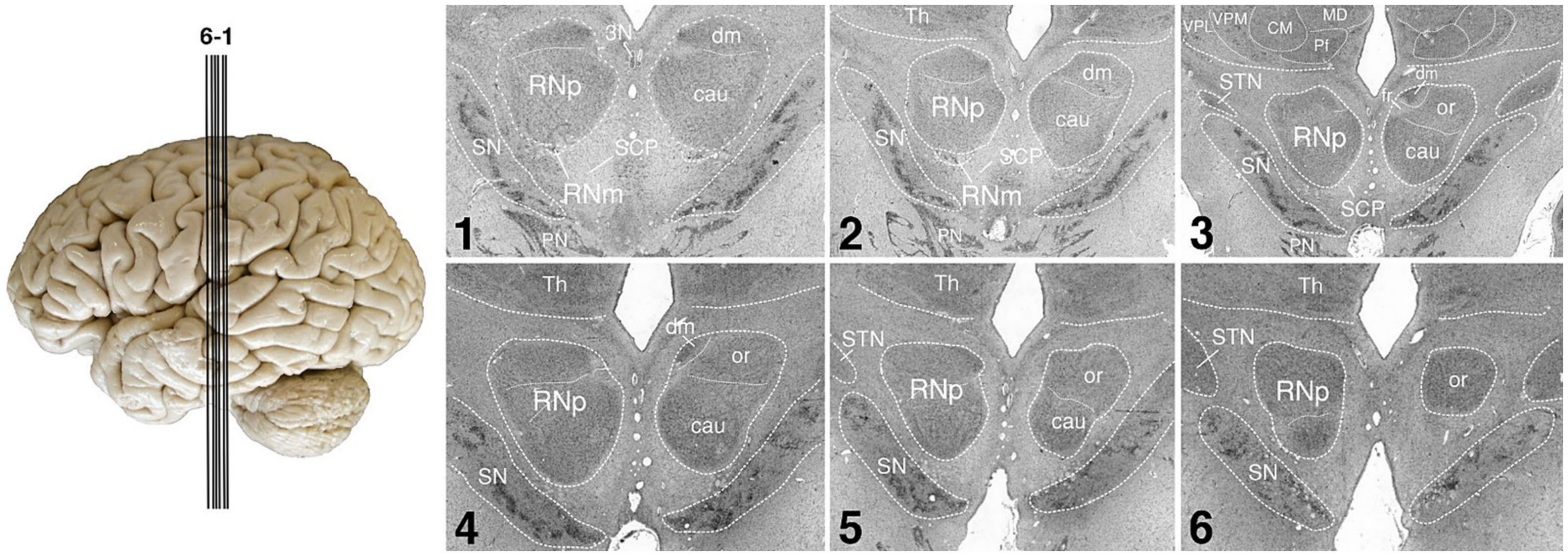
Human RN. The figure shows the human RN in a series of coronal silver-stained sections. The RN is shown in the left and right hemisphere. The two main subdivisions of RN, the RNp and RNm are shown in the left hemisphere, further possible parcellation of RNp is indicated in the right hemisphere. Note that the RNm is small and only visible in the first two of the depicted sections. The sections 1–6 are arranged from caudal to rostral. The approximate position of each section is indicated by the lines on the brain left. SN, substantia nigra; cau, caudal part of the RNp; CM, centromedian nucleus of the thalamus; dm, dorsomedial part of the RNp; fr, fasciculus retroflexus; or, oral part of the RNp; MD, mediodorsal nucleus of the thalamus; Pf, parafascicular nucleus of the thalamus; PN, pontine nuclei; RNm, magnocellular part of the red nucleus; RNp, parvocellular part of the red nucleus; SCP, superior cerebellar peduncle; STN, subthalamic nucleus; Th, thalamus; VPL, lateral part of the ventral posterior nucleus of the thalamus; VPM, medial part of the ventral posterior nucleus of the thalamus; 3N, oculomotor nucleus.

On the other hand, the rubro-olivary projection within the central tegmental tract is considered large in humans (Massion, 1967; Nathan and Smith, 1982; Onodera and Hicks, 2009; Basile et al., 2021; Olivares-Moreno et al., 2021). It is possible that due to the expansion of the cortical input to RN and the emergence of neocerebellum, the RNp increased in size during the evolution of humans and possibly acquired different, perhaps more sensory or even cognitive functions (Kennedy et al., 1986; ten Donkelaar, 1988; Liu et al., 2000; Nioche et al., 2009; Gruber and Gould, 2010; Habas et al., 2010). The RNp is large in humans and can be further subdivided into oral, caudal and dorsomedial parts (Figure 2; Pu et al., 2000; Büttner-Ennever et al., 2014; see also Onodera and Hicks, 2009).

Despite the well-known inter-species differences, systematic comparative studies of RN remain rare. However, a direct comparison of RN across a representative variety of primate species is crucial to capture the evolutionary changes that shaped the RN. Understanding the variability of the RN structure between species in combination with species-specific motor skills and adaptations might enhance our functional understanding of the RN circuitry in humans as well. Moreover, studies of our own group have shown that the anatomy of human brains varies, while the degree of variability depends on the brain region. This makes it necessary to analyze several human brains, which then provide the basis to compute probabilistic maps in stereotaxic space (Zilles et al., 2013). These maps then can also serve for comparison with *in vivo* neuroimaging studies including fMRI and dMRI. We therefore delineated RNm and RNp in serial histological sections of 20 primate species over their full extent in order to further scrutinize the evolution of the RN and to provide human cytoarchitectonic probabilistic maps for the Julich-Brain (Amunts et al., 2020) as well as delineations in the microscopic BigBrain (Amunts et al., 2013).

## Materials and methods

### Specimen and tissue preparation

#### Human brains

Eleven human *post-mortem* brains (six male and five female, Table 1) were used in this study. All brains were obtained from the body donor program of the Department of anatomy at the University of Düsseldorf in accordance to the rules of the local ethics committee (#4863). For each brain there is a protocol containing the fresh brain weight that can be used to estimate the fresh brain volume (Amunts et al., 2005). Ten brains were employed to compute cytoarchitectonic probability maps (see below) and one brain provided reference data in the 3D BigBrain model at high resolution of 20 μm isotropic (Amunts et al., 2013). The BigBrain model serves as a microscopical template in the Human Brain Atlas of EBRAINS,^1^ the research infrastructure created by the Human Brain Project (Amunts et al., 2019).

**Table 1:** Overview of postmortem human brains.

ID	Section type	Gender	Age	Fresh brain weight (g)
pm15	Horizontal	Male	54	1,260
pm16	Horizontal	Male	63	1,340
pm6	Coronal	Male	54	1,622
pm7	Coronal	Male	37	1,437
pm11	Coronal	Male	74	1,381
pm5	Coronal	Female	59	1,142
pm8	Coronal	Female	72	1,216
pm9	Coronal	Female	79	1,110
pm10	Coronal	Female	85	1,046
pm12	Coronal	Female	43	1,198
pm20*	Coronal	Male	65	1,392

*High-resolution brain model BigBrain (Amunts et al., 2013).

Each brain was immersion fixed with 4% formalin or Bodian solution (a mixture of formalin, glacial acetic acid and alcohol). Brains were embedded in paraffin and serially sectioned with a thickness of 20 μm along the horizontal or coronal planes. Every 15th section (every section in the BigBrain, i.e., 7,404 coronal sections) was mounted onto a gelatin-covered glass slide, corresponding to 300 μm distance between mounted sections in the human brain sample. The brain sections were stained with a modified silver method for cell bodies (Merker, 1983).

#### Non-human brains

Additionally, 25 non-human *post-mortem* brains were obtained from the brain collection at the C. & O. Vogt Institute for Brain Research of the Heinrich Heine University in Düsseldorf (Zilles et al., 2011). The postmortem delay was less than 12 h. These specimens comprised 19 different non-human primate species and were used for an evolutionary comparison of the red nucleus (Table 2). The fresh brain weights of the species were calculated based on the fixed brain weight using correction factors (Amunts et al., 2005). The fresh brain weight was then used to calculate the fresh volume. The individual shrinkage factors were determined by the ratio between the estimated fresh brain volume and volume after histological processing.

**Table 2:** Overview of postmortem non-human primate brains.

Taxon	Species	Name	Sex	Section plane	Staining
Hominidae	*Hylobates lar*	YN81-146	f	Coronal	Merker
Hominidae	*Hylobates lar*	3_97	NA	Coronal	Merker
Hominidae	*Pan troglodytes*	1,548	NA	Coronal	Merker
Hominidae	*Pan troglodytes*	YN89-278	m	Horizontal	Merker
Hominidae	*Pan troglodytes*	4_97	f	Coronal	Merker
Hominidae	*Pan paniscus*	YN86-137	f	Coronal	Merker
Hominidae	*Pan paniscus*	1_97	f	Coronal	Merker
Hominidae	*Pongo pygmaeus*	2_97	m	Coronal	Merker
Hominidae	*Pongo pygmaeus*	YN 85–38	m	Horizontal	Merker
Hominidae	*Pongo pygmaeus*	5_97	m	Coronal	Merker
Hominidae	*Gorilla gorilla*	YN82-140	f	Coronal	Merker
Cercopithecidae	*Macaca fascicularis*	P24	NA	Coronal	Nissl
Cercopithecidae	*Macaca mulatta*	DP1	NA	Coronal	Merker
Cercopithecidae	*Lophocebus albigena*	A221	m	Coronal	Merker
Cercopithecidae	*Cercopithecus Spec.*	Sixi	NA	Coronal	Nissl
Platyrrhini	*Aotus trivirgatus*	888	f	Coronal	Nissl
Platyrrhini	*Saimiri sciureus*	2,408	m	Coronal	Merker
Platyrrhini	*Callithrix jacchus*	1,091	NA	Coronal	Nissl
Prosimiae	*Lemur catta*	1,514	f	Coronal	Nissl
Prosimiae	*Lepilemur ruficaudatus*	M81	f	Coronal	Nissl
Prosimiae	*Indri indri*	M192	f	Coronal	Nissl
Prosimiae	*Daubentonia madagascariensis*	M53	f	Coronal	Nissl
Prosimiae	*Nycticebus coucang*	1966/44	f	Coronal	Nissl
Prosimiae	*Galago senegalensis*	A338	f	Coronal	Nissl
Prosimiae	*Tarsius bancanus*	1,333	f	Coronal	Nissl

The family and species name, brain code, as well as sectioning and staining type. Sections are stained using silver staining for cell bodies according to Merker (1983) or Nissl staining with cresyl-violet.

We categorized all data into 5 groups of primates: “humans” (*n* = 10), “non-human apes” (i.e., lesser and great apes excluding humans) (*n* = 11), “old world monkeys” (Cercopithecidae, i.e., old world monkeys in a restricted sense) (*n* = 4), “new world monkeys” (Platyrrhini) (*n* = 3) and “prosimians” (Prosimiae) (*n* = 7). For a detailed list of all species included within these groups see Table 3 and Figure 1. The brain sections were stained with a modified silver method for cell bodies (Merker, 1983) or with Nissl.

**Table 3:** Mean values of brain and RN volume in the five different groups (*Homo*, *Hominidae*, *Cercopithecidae*, *Platyrrhini*, and *Prosimiae*).

Taxon	Species	BV fresh (cm^3^)	RNp right (mm^3^)	RNp left (mm^3^)	RNm right (mm^3^)	RNm left (mm^3^)	*n*
Homo	*Homo sapiens*	1235.7	289.9	293.1	3.1	3.0	10
Hominidae	*Gorilla gorilla*	98.8	33.7	34.3	2.6	2.0	1
Hominidae	*Hylobates lar*	396.8	131.1	129.3	6.8	7.4	2
Hominidae	*Pan paniscus*	354.8	141.3	124.5	7.9	6.0	2
Hominidae	*Pan troglodytes*	368.9	100.2	101.7	4.4	4.9	3
Hominidae	*Pongo pygmaeus*	364.3	123.7	127.9	1.5	3.0	3
Cercopithecidae	*Lophocebus albigena*	53.6	8.7	9.9	7.6	7.7	1
Cercopithecidae	*Cercopithecus Spec.*	90.1	19.0	18.6	7.1	7.9	1
Cercopithecidae	*Macaca fascicularis*	84.3	33.6	35.6	15.1	13.8	1
Cercopithecidae	*Macaca mulatta*	64.9	10.9	11.8	6.3	6.1	1
Platyrrhini	*Aotus trivirgatus*	16.9	1.5	1.8	4.6	4.3	1
Platyrrhini	*Callithrix jacchus*	15.2	1.6	1.5	3.1	2.8	1
Platyrrhini	*Saimiri scuireus*	9.0	0.3	0.2	1.3	1.4	1
Prosimiae	*Daubentonia madagescariensis*	22.6	2.5	3.2	5.6	5.3	1
Prosimiae	*Galago senegalensis*	8.0	0.3	0.3	2.2	2.4	1
Prosimiae	*Indri indri*	36.4	2.8	2.8	4.9	4.8	1
Prosimiae	*Lemur catta*	46.7	6.8	8.3	6.5	6.3	1
Prosimiae	*Lepilemur ruficaudatus*	12.1	0.9	0.9	2.8	2.7	1
Prosimiae	*Nycticebus coucang*	2.8	0.8	0.7	1.1	1.1	1
Prosimiae	*Tarsius bancanus*	3.0	0.9	0.8	0.7	0.8	1

The total brain volume (BV), as well as the RNp and RNm volumes of the left and right hemisphere are shown. All volumes were corrected for shrinkage.

### Cytoarchitectonic mapping of the RNm and RNp

**Figure 3:**
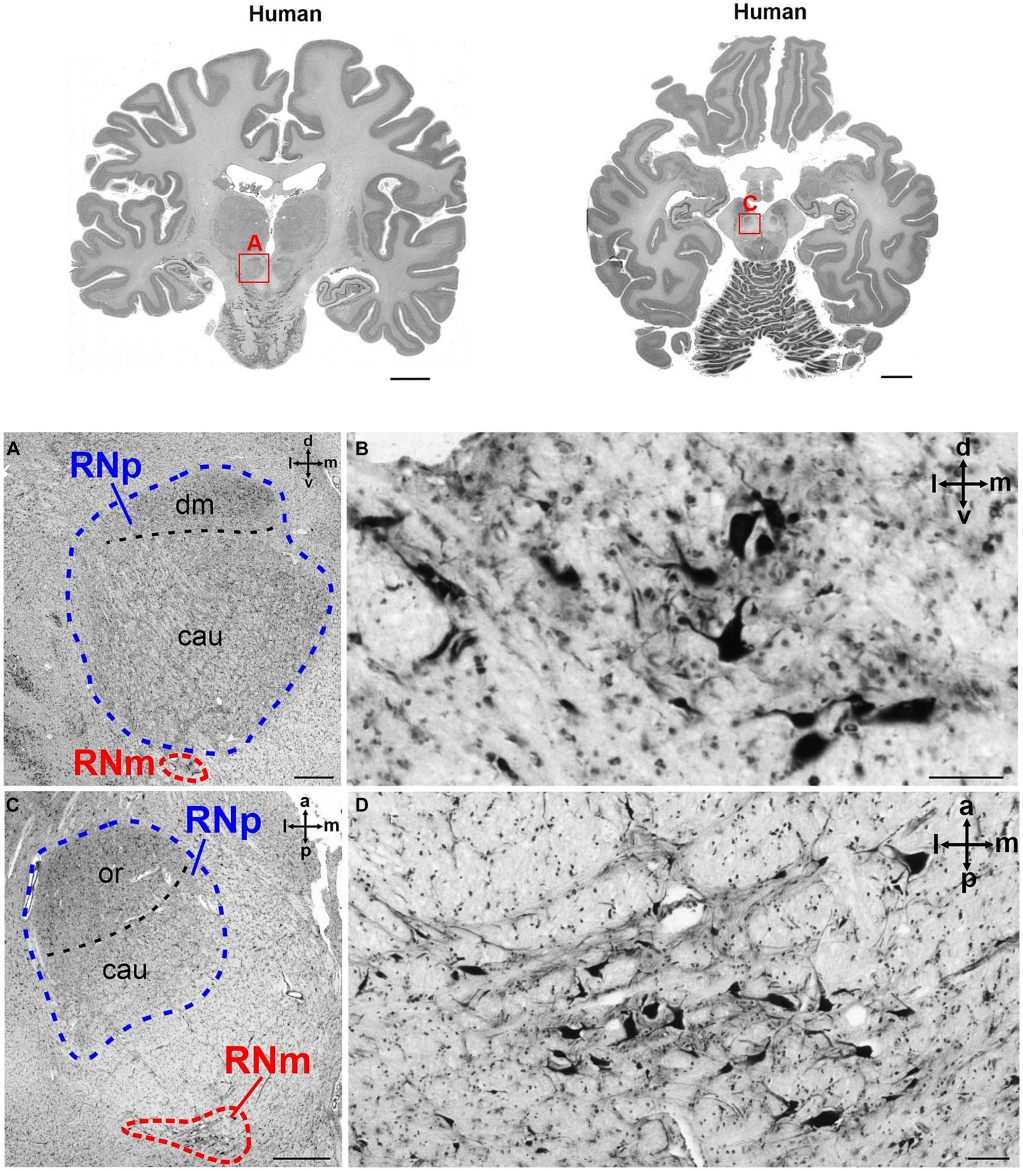
Cytoarchitecture of the human RN (*Homo sapiens*, family *Hominidae*). The upper part of the figure shows two human brain slices in coronal (left) and horizontal (right) plane. The RN is marked with a red rectangle in both slices and magnified in **(A,C)**, respectively. **(A)** Magnified region marked with A in the brain slices above showing the RNp (blue) and a small RNm (red) juxtaposed ventrally to the caudal RNp. The cell density varies slightly across the RNp and allows the subdivision into the dorsomedial (dm) and caudal (cau) part. **(B)** High magnification of the RNm shown in A. **(C)** Higher magnification of the region marked with C in the brain slice above showing a horizontal section through the RNp (blue) and RNm (red). In this case the depicted RNm cluster is not juxtaposed to the RNp but is located more caudally. **(D)** High magnification of the RNm cluster shown in RNm. cau, caudal part of the RNp; dm, dorsomedial part of the RNp; or, oral part of the RNp; RNm, magnocellular part of the red nucleus; RNp, parvocellular part of the red nucleus. The subdivision of the RNp into dorsomedial (dm), oral (or) and caudal (cau) portions is based on Büttner-Ennever et al. (2014) (see also Figure 2). Scale bar: 1 cm next to the slices, 1 mm in A and C and 100 μm in **(B,D)**.

The two subdivisions of the red nucleus were identified by large neurons with a polygonal or multipolar perikaryon in the RNm, and smaller neurons with a triangular or ovoid perikaryon in the RNp in accordance to the literature (Kuypers and Lawrence, 1967; King et al., 1971; Padel et al., 1981; Burman et al., 2000a,b; Onodera and Hicks, 2009, 2010; Büttner-Ennever et al., 2014). We subdivided the RN into the RNm and RNp (Figures 2, 3). It should be noted however, that further parcellations are possible. Previously, the human RNp has been further subdivided into oral, caudal and dorsomedial parts (Pu et al., 2000; Büttner-Ennever et al., 2014; see Figure 2) or into ventrolateral, dorsomedial and a part regarded as the nucleus accessorius medialis of Bechterew (NB) (Onodera and Hicks, 2009).

The mapping of the RNp and RNm boundaries was performed in serial sections of both hemispheres in high-resolution images (20 μm and 1 μm in-plane resolution) of histological sections using in-house software (OnlineSectionTracer, Amunts et al., 2020). Every 60th to 15th section has been analyzed, i.e., distances between sections were 1.2 mm to 0.3 mm. The Atelier 3D software (A3D, National Research Council of Canada, Canada, Borgeat et al., 2007) was used for the 3D reconstruction of the RN in the BigBrain data set. The volumes of the RN and its two subdivisions were calculated based on section thickness, distance between the measured sections, and shrinkage factor (Amunts et al., 2005, 2007).

### Three-dimensional reconstruction and probabilistic maps of the red nucleus in the human brain

The digitized histological sections and delineated nuclei were 3D reconstructed in each post-mortem brain (Amunts et al., 2020). In short, the 3D reconstruction was corrected for distortions that occurred during histological processing (embedding, sectioning and mounting). Then the RN of the ten 3D-reconstructed brains were spatially normalized to two template spaces, which are frequently used in the neuroimaging community: the single subject reference template of the Montreal Neurological Institute, MNI-Colin27 and the non-linear asymmetric MNI152 2009c template space (ICBM152casym; Evans et al., 2012). The spatial resolution of the maps in these two templates is 1 mm isotropic.

The delineations of the RN in the ten brains were superimposed in each hemisphere to compute a probability map of the RN. This map captures variations in size, shape and localization and provides information about the probability an individual RN can be found at a certain position in the reference brain. The spatial resolution of the maps in these two templates is 1 mm isotropic.

### Statistical procedure

For the analysis and figures Matlab (R2022b) and RStudio (2022.12.0; R software version 4.2.2) were used. Normal distribution was checked with the Anderson-Darling test for parametric tests. The phylogenetical tree of primates was downloaded from the 10kTrees website https://10ktrees.nunn-lab.org (Arnold et al., 2010). The data on cerebellum volume were included from Navarrete et al. (2018), Stephan et al. (1981), and Akeret et al. (2021).

Log-transformed means of region and brain volumes were used in the allometric scaling analysis of the RN and its subnuclei. Because comparative data is not expected to be independent due to their shared phylogenetic history, we used regression procedures that account for phylogenetic relatedness. Specifically, we assessed the scaling relationship using phylogenetic generalized least-squares (pGLS; Rohlf, 2001). To investigate the evolutionary history of species’ deviations from allometry, we used species‘residual deviation from the allometry in a multi-regime Ornstein-Uhlenbeck (‘OU‘) modeling (Butler and King, 2004). This approach considers comparative data in conjunction with a phylogenetic tree and estimates when comparative differences have arisen along branches of the phylogeny. Considering that we used residual deviations from allometry, estimated differences in mean trait value are representative of differences in allometric intercept. This procedure estimates comparative differences in mean trait value (intercept) directly from the data and the tree (i.e., without any *a priori* hypothesis as to which species, or group of species, exhibits a difference in mean trait value). The OU modeling hereby quantifies the evolution of a continuous trait ‘X’ as dX(t) = *α*[*θ* – X(t)]dt + *σ*dB(t) where ‘*σ*’ captures the stochastic evolution of Brownian motion (BM), ‘*α*’ determines the rate of adaptive evolution toward an optimum trait value ‘*θ*’. Here we use OU modeling to identify when shifts in the residual size of RNp and RNm relative to brain size occurred in the evolutionary history of primates. The uncertainty of estimating patterns of evolutionary history is ubiquitous but can be partly overcome by quantifying uncertainty. One way to do so in the context of OU modeling is to quantify effect size (which is proportional to power). Here we use the signal-to-noise ratio ($\sqrt{\eta }\phi$) as proposed by Cressler et al. (2015). When $\sqrt{\eta }\phi \gg 1$ effect size is high and we can be confident that the obtained results are accurate. OU modeling was implemented using the ‘l1ou’ (Khabbazian et al., 2016) package in the R software environment.

To validate the evolutionary hypothesis estimated by multi-regime OU modeling, we translated the estimated hypothesis to a least-squares framework and tested it using least-squares phylogenetic analysis of covariance (pANCOVA; Smaers and Rohlf, 2016). The pGLS and the pANCOVA were implemented using the ‘evomap’ (Smaers, 2014) package in the R software environment.

To map the evolutionary diversification of residual size of RNp and RNm relative to brain size we used ancestral state estimation (‘ASE’). ASE estimates nodal values based on observed variation and phylogenetic structure. ASE estimates point estimates for each ancestral node, and hereby differs from OU modeling which typically only estimates the phylogenetic location of large shifts in trait values. The uncertainty in the estimation of point estimates of nodal values is inherently high, and therefore ASE should only be used for visualizing trends through time (Smaers and Mongle, 2017). Here we use the multiple variance BM (‘mvBM’) approach proposed by Smaers et al. (2016), because it accounts for different rates of evolution along different branches when estimating ancestral values. The mvBM was also implemented using the ‘evomap’ (Smaers, 2014) package in the R software environment.

## Results

### Cytoarchitecture of the red nucleus

**Figure 4:**
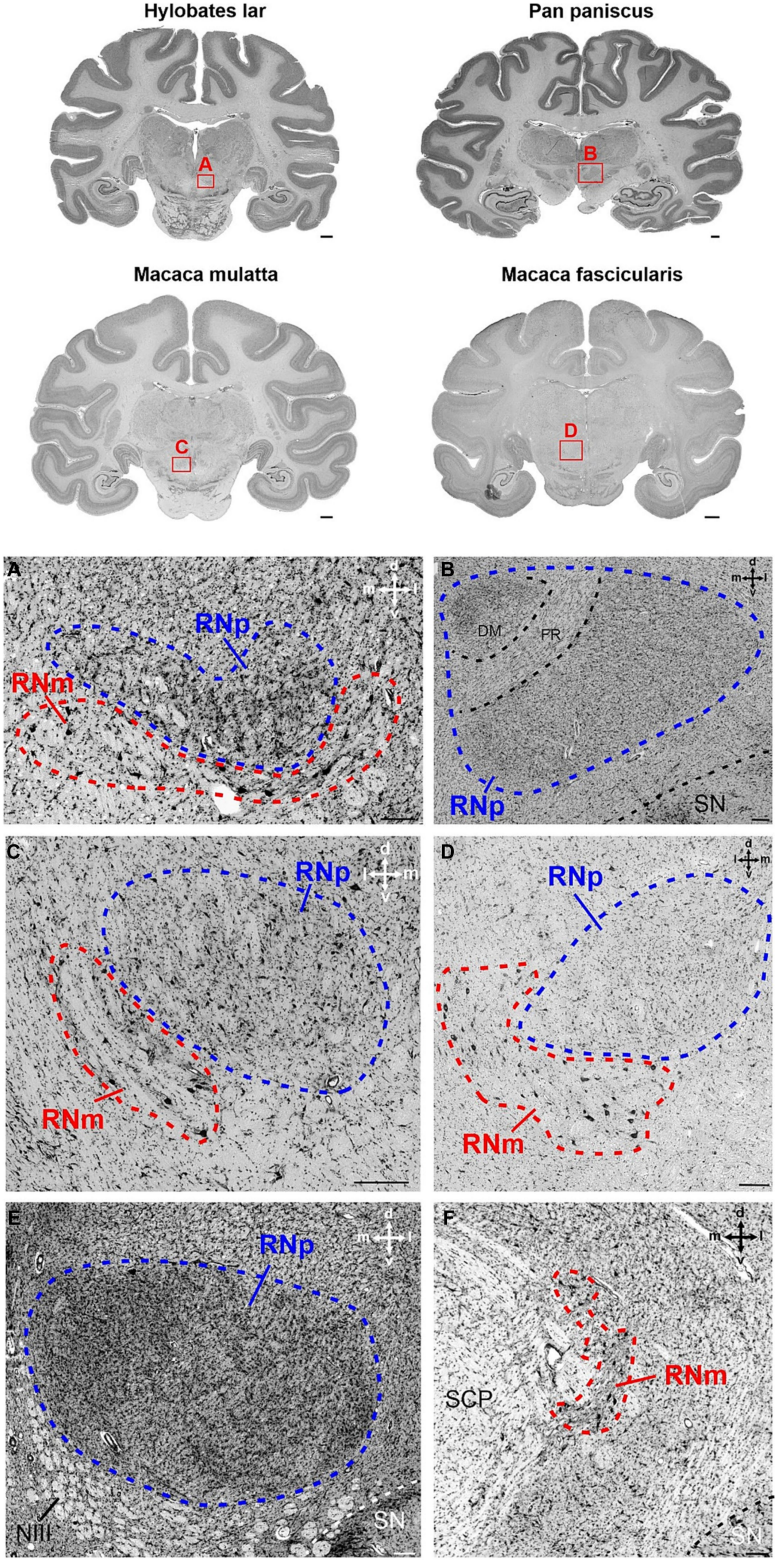
Cytoarchitecture of the red nucleus in non-human apes and old world monkeys. Brain slices of four different species are shown in the upper part of the figure. *Hylobates lar* (gibbon) and *Pan paniscus* (bonobo) represent two examples from the group of non-human apes. In the second raw, the brain sections of *Macaca mulatta* (rhesus monkey) and *Macaca fascicularis* (crab-eating macaque) from the group of old world monkeys are depicted. The red rectangles with the letters A–D indicate the region magnified in the corresponding panels **(A–D)**. **(A)** Section showing the posterior part of the RNp ventrally attached by the anterior RNm in *Hylobates lar*. Note the low density of large neurons in RNm as compared to more densely packed medium-sized neurons within the RNp. **(B)** Magnified region of the RNp in *Pan paniscus* showing the dorsomedial aspect of the RNp that shows slightly higher cell density and is encapsulated by the fasciculus retroflexus (FR). The section was chosen at the level, where FR is largest, such that the separation of the dorsomedial region is most pronounced. Note also the irregular cell distribution within the rest of the RNp. **(C)** Magnified region of caudal RNp and rostral RNm from *Macaca mulatta*. The RNm is juxtaposed ventrolaterally to the caudal RNp. The RNm displays loosely scattered large neurons, while neurons in RNp are slightly smaller and more densely packed. Note polygonal, fusiform and round cells within the RNp. **(D)** Magnified region of the RNp and RNm in *Macaca fascicularis*. The section is chosen at the caudal level of RNp to show the ventrolateral attachment of the anterior RNm. Note scattered large/giant neurons interspersed with smaller neurons within the RNm. Neurons in RNp are medium-sized and more densely packed. **(E)** RNp from *Hylobates lar* showing high but variable density within the RNp. The density increases especially toward the medial pole of the RNp but also toward the lateral rim of the nucleus. **(F)** RNm in *Pan paniscus* in a more caudal section of the same animal as in B. The RNm is formed by loosely packed large neurons in the dorsomedial vicinity of the fibers of the superior cerebellar peduncle SCP. The cross in the right corner of the panels **(A–F)** indicates the orientation in the dorsal (d), ventral (v), medial (m) and lateral (l) direction. DM, dorsomedial; FR, fasciculus retroflexus; NIII, oculomotor nerve; RNm, magnocellular part of the red nucleus; RNp, parvocellular part of the red nucleus; SCP, superior cerebellar peduncle; SN, substantia nigra. Scale bars next to the overview brain slices represent 2 cm and 200 μm in **(A–F)**.

**Figure 5:**
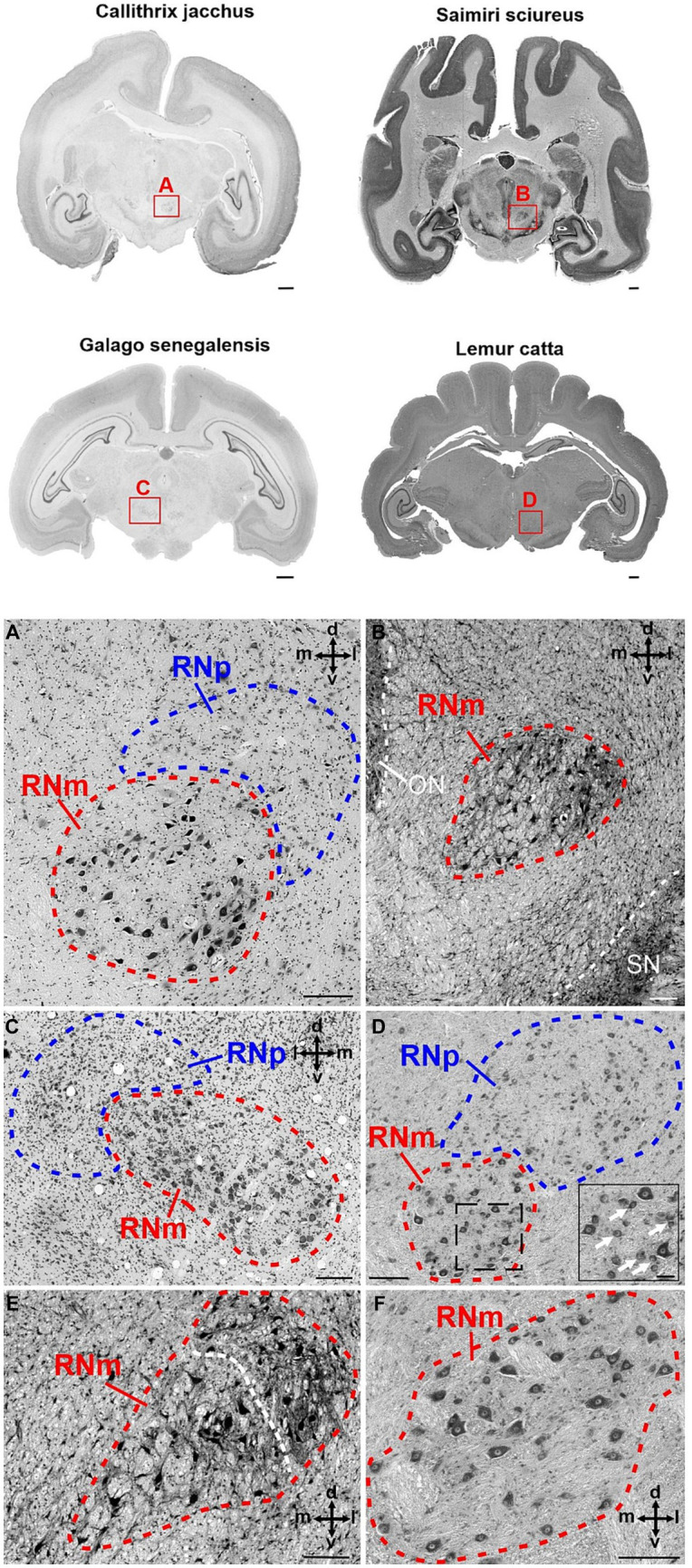
Cytoarchitecture of the red nucleus in new world monkeys and prosimians. The upper part of the figure displays brain slices of four different species. *Callithrix jacchus* (common marmoset) and *Saimiri sciureus* (common squirrel monkey) represent two examples from the group of new world monkeys, while *Galago senegalensis* (Senegal bushbaby) and *Lemur catta* (ring-tailed lemur) both belong to the group of prosimians. The red rectangles with the letters A–D indicate the region magnified in the corresponding panels **(A–D)**. **(A)** Higher magnification of the RNp and RNm in *Callithrix jacchus*. The RNp lies dorsolaterally to the RNm. RNm has a round shape filled with large/giant neurons often with a neuron-free protuberance in the middle. **(B)** Magnified RNm region in *Saimiri sciureus*. RNm has round to oval shape and is formed by many large/giant neurons with relatively large spaces between the somata of the cells. The density slightly increases toward the dorsolateral aspects, where also smaller neurons are present. This is especially pronounced in the more anterior part of RNm as shown in **(E)**. **(C)** Higher magnification of the RN in *Galago senegalensis*. RNm is oval in shape, ventromedially adjacent to the RNp and consists of densely packed large neurons. Some spots with low cell density are also present. **(D)** The panel shows the anterior portion of the RNm with dorsolaterally juxtaposed RNp in Lemur catta. The RNm consists of loosely packed large/giant neurons interspersed with smaller round and oval neurons (see insert). The dashed lined rectangle indicates the magnified region of the RNm in the insert. The white arrows in the insert point to examples of smaller neurons distributed in-between the large neurons. The occurrence of such smaller neurons within the RNm is pronounced in the anterior pole of the RNm while such small neurons are virtually absent in more caudal sections of the RNm [see panel **(F)**]. **(E)** RNm section in *Saimiri sciureus* anterior to the section presented in **(B)**. The cell density increases while the cell size decreases toward the dorsolateral aspect of the RNm (indicated by the white dashed line). **(F)** High magnification of the RNm *Lemur catta* posterior to the section shown in **(D)**. In contrast to the anterior RNm small neurons are virtually absent here. The cross in the panels indicates the orientation in the dorsal (d), ventral (v), medial (m) and lateral (l) direction. ON, oculomotor nucleus; RNm, magnocellular part of the red nucleus; RNp, parvocellular part of the red nucleus; SN, substantia nigra. Scale bars next to the overview brain slices represent 1 cm, 200 μm in **(A–F)** and 50 μm in the insert in **(D)**.

Although the size of RNp and RNm differed profoundly between species (see below), the cytoarchitecture varied rather slightly and the general picture was comparable in all primates (Figures 3–5). The RNp consisted mainly of medium-sized neurons mostly with a polygonal soma shape but round and fusiform somata were also frequently observed (Figures 4A–E, 5A,B,D). The density of neurons varied across the RNp giving it a mosaic-like appearance, however the extent to which the density varied differed across species (Figures 4A–E). In general, the cell density was usually lowest in the center of RNp and increased toward the medial, dorsomedial or ventrolateral direction. In some specimens such as in *Hylobates lar*, there was a tendency for a much higher cell density toward the medial pole of RNp (Figure 4E). In others such as *Pan paniscus* or *Pan troglodytes*, a small dorsomedial subdivision was apparent due to increased cell density and encapsulation by fibers running underneath this subdivision through the RNp (Figure 4B). It should be noted that in some species (e.g., prosimians: *Nycticebus coucang, Daubentonia madagascariensis, Lepilemur ruficaudatus* or new world monkey: *Callithrix jacchus*) the RNp was much less discernable compared to other species, especially those with large RNp (Figure 5).

Caudally to RNp, we observed large/giant neurons forming the RNm. The size of RNm was subjected to high variations. It was generally smaller and less pronounced in the *Hominoidae* superfamily (Figures 4A,F) and larger in the monkeys (*Cercopithecidae, Platyrrhini*, *and Prosimiae*) (Figures 4C,D, 5). In *Hominoidea*, the RNm consisted mostly of few scattered large neurons adjacent dorsally to fibers of the superior cerebellar peduncle (SCP) especially in its caudal extension, around the level of its decussation (Figure 4F). In species with well-developed RNm, this cell group extended further rostrally and formed round or oval easily discernable nuclei with polygonal, round and some oval or elongated neurons (Figures 4A,C, 5A–F). The anterior aspect of RNm either protruded ventrally (Figure 4A), ventrolaterally (Figure 4C) or ventromedially (Figures 4, 5A,C) to caudal RNp or was only loosely attached to caudal RNp, occasionally with a small gap in-between. The anterior RNm sometimes contained large neurons that were slightly more densely packed than the giant neurons. Such an agglomeration of large (but smaller than giant) neurons was seen for example in *Saimiri sciureus* in the dorsolateral aspect of the anterior RNm (Figure 5E). In *Lemur catta*, the anterior aspect of RNm was interspersed with small neurons with oval and round shaped cell bodies (Figure 5D).

### 3D maps of the human red nucleus

The RNp in humans consisted mainly of medium-sized neurons with variable rather loose packing density (Figures 3A,C), while the RNm showed scattered groups of large neurons (Figures 3B,D). The RNp was often interspersed with fibers including those belonging to the SCP, the fasciculus retroflexus and the oculomotor nerve (Figure 2). The RNm was less homogeneous than the RNp, and often consisted of only few clusters with a handful of neurons on the mapped sections. These clusters spanned on average 1.9 mm along the anterior–posterior extent and were either attached directly to the caudal RNp (Figure 3A) or were found further caudally (Figure 3C).

**Figure 6:**
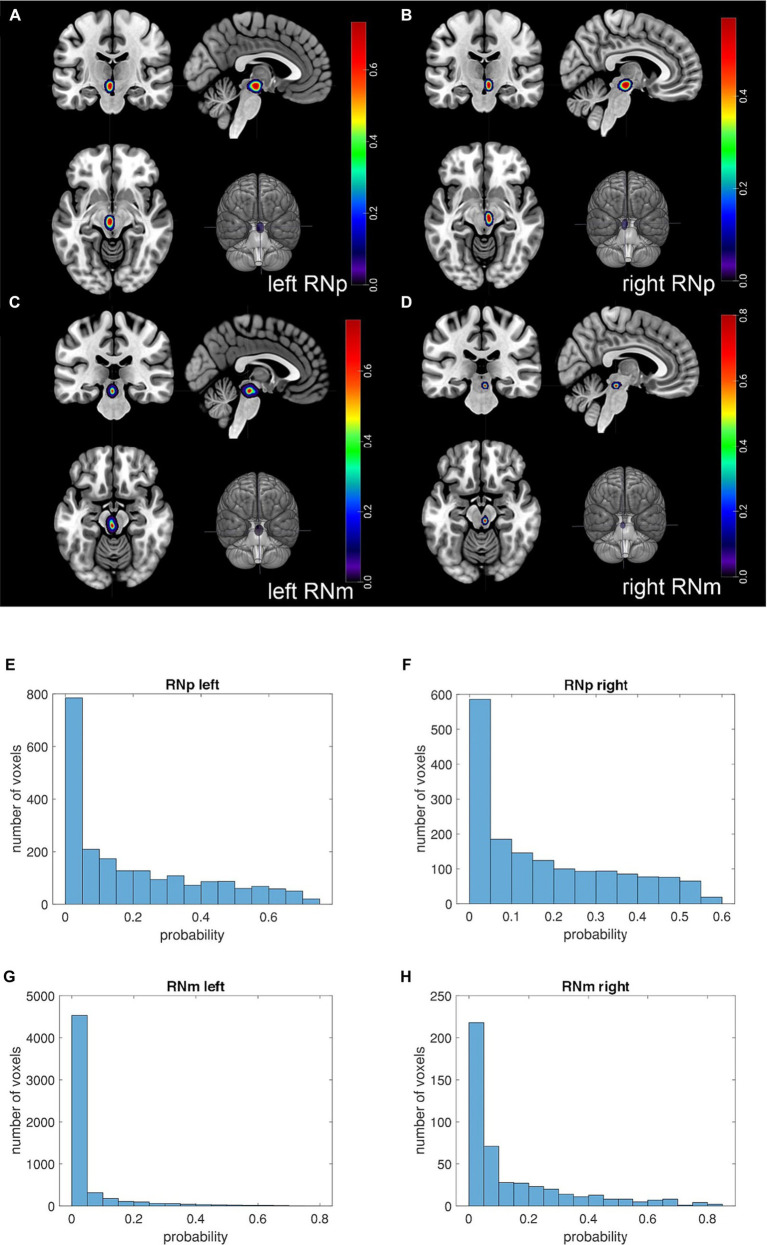
Probability maps (p-maps) of the RN in the single subject MNI template. Figure depicts the p-maps of the RN based on 10 subjects. The p-maps provide for each voxel the probability of the RN being present in the given voxel. **(A–D)** The location of the respective p-map is shown in a coronal (upper left corner), sagittal (upper right corner) and horizontal (lower left corner) plane as well as its location in a rendered brain (lower right corner). **(A)** The panel shows the p-map of the left RNp. **(B)** The panel shows the p-map of the right RNp. **(C)** The panel shows the p-map of the left RNm. **(D)** The panel shows the p-map of the right RNm. The color coding indicates the probability of the RN being present in the voxel. Note that the slices in the panels **(A–D)** are selected to show the cross-section of the highly probable location of the nucleus. Thus, many voxels in these sections display higher probability (>0.4), especially in RNp **(A,B)**. However, toward the borders of the p-maps the probability decreases and the majority of the voxels display low probabilities. This is illustrated in the histograms below **(E–H)** showing that only a fraction of the voxels of the p-map shows higher probabilities. The RNp maps appear quite comparable between hemispheres **(A vs. B)** and the total number of voxels differs only slightly **(E vs. F)** (total number of voxels left RNp = 2,124; right RNp = 1,650). On the other hand, the p-map of the left RNm appears larger than that of the right RNm **(C vs. D)** and consequently the total number of voxels of the left RNm map is considerably higher than that of the right RNm [**(G vs. H)**; total number of voxels left RNm = 5,509; right RNm = 468]. Nevertheless, the majority of the voxels represents probabilities below 10% **(G,H)** indicating interindividual variability.

Based on the RN delineations in the ten brains (Table 1) 3D probability maps (Figures 6A–D) (in MNI-Colin27 and ICBM152casym) were calculated (see Methods). The maps quantify the probability of observing a particular nucleus at a specific stereotaxic location. The RNp showed a low inter-subject variability in both hemispheres (Figures 6A,B). The variability of the RNm was very high. While the maximum probability in RNp was 99.8%, it reached only 17.5% in RNm. Further, the RNp maps appear quite comparable between hemispheres (Figures 6A,B) and the total number of voxels differs only a little (total number of voxels left RNp = 2,124; right RNp = 1,650; Figures 6E,F). In contrast, the p-map of the left RNm is larger (Figures 6C,D) and consequently the total number of voxels of the left RNm map is considerably higher than that of the right RNm (total number of voxels left RNm = 5,509; right RNm = 468; Figures 6G,H). However, the majority of the voxels represents probabilities below 10% and the volume analysis did not reveal any differences between the left and right RNm (*p* < 0.05, see below).

**Figure 7:**
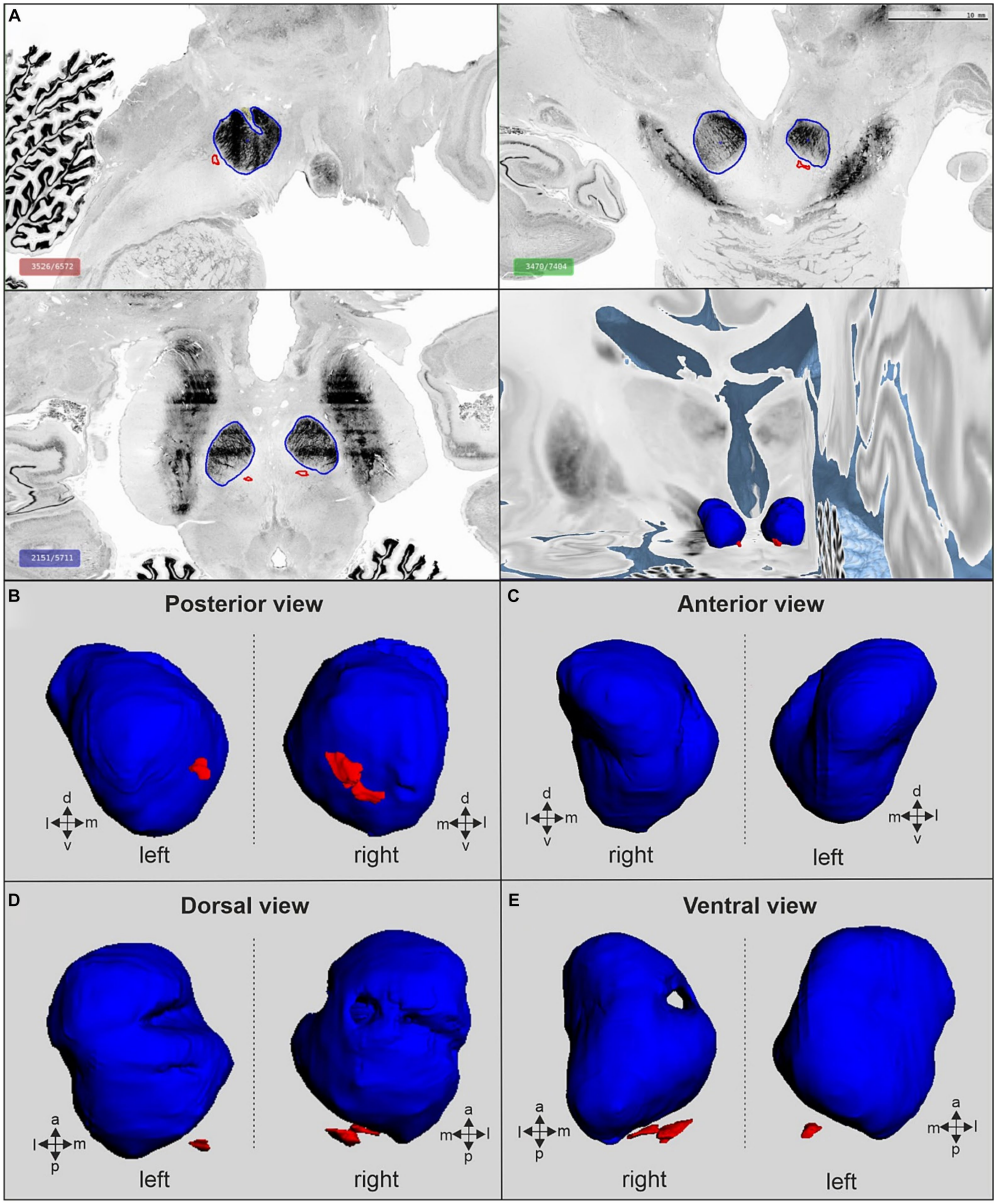
The red nucleus in the BigBrain space. **(A)** Red nucleus in sagittal (top left), coronal (top right) and horizontal (bottom left) cutting planes and corresponding 3D-reconstruction from posterior view (bottom right). **(B)** 3D view of the RN from posterior. **(C)** 3D view of the RN from the rostral site. **(D)** 3D reconstruction of the RN from the dorsal view. **(E)** Ventral view of the 3D reconstruction of the RN. The parvocellular (RNp) and the magnocellular (RNm) are labeled by blue and red, respectively. Data are available through the interactive atlas viewer of the BigBrain template in the HBP Human Brain Atlas at www.ebrains.eu.

The 3D reconstruction of RNm and RNp in the BigBrain model (brain 11, Table 1) revealed a small dimple in the RNp of the left hemisphere (Figure 7). The RNp was crossed by a thin fiber bundle in the right hemisphere. The RNm was located posteriorly to the RNp and consisted of clusters of large cells that are separated from the RNp. The mapping data are available in the Human Brain Atlas of EBRAINS (see Footnote 1), where they can be visualized and analyzed in the context of other brain structures and fiber tracts. They can also be explored and downloaded via the Julich-Brain Cytoarchitectonic Atlas viewer.^2^

### The volume of the red nucleus across primates

The total bilateral volume of the red nucleus in humans was on average 589.10 mm^3^ ± 54.69 mm^3^. The left and right RN did not differ between each other in volume (left: 296.05 mm^3^ ± 28.09 mm^3^, right: 293.05 mm^3^ ± 26.79 mm^3^, Wilcoxon signed rank test, *p* > 0.05). Similarly, the two cytoarchitectonic RN subdivisions did not differ between the two hemispheres (RNp: Wilcoxon signed rank test, *p* > 0.05, RNm: paired t-test, *p* > 0.05), however, the RNp was considerably larger than RNm (582.99 mm^3^ ± 54.56 mm^3^ vs. 6.11 mm^3^ ± 0.60 mm^3^, respectively).

The RN volume in non-human species was smaller than in humans (non-human apes: 219.83 mm^3^ ± 29.20 mm^3^; old world monkeys: 54.95 mm^3^ ± 15.03 mm^3^; new world monkeys: 8.15 mm^3^ ± 2.65 mm^3^; prosimians: 11.34 mm^3^ ± 3.43 mm^3^) with no significant differences between the left and right hemisphere (Wilcoxon signed rank test, *p* > 0.05 for all left vs. right within group comparisons of total RN volume, RNp volume and RNm volume). Note that our sample size in the old and new world monkey groups might be rather small for revealing a putative asymmetry effect (*n* = 4 and *n* = 3, respectively, see Table 3).

**Figure 8:**
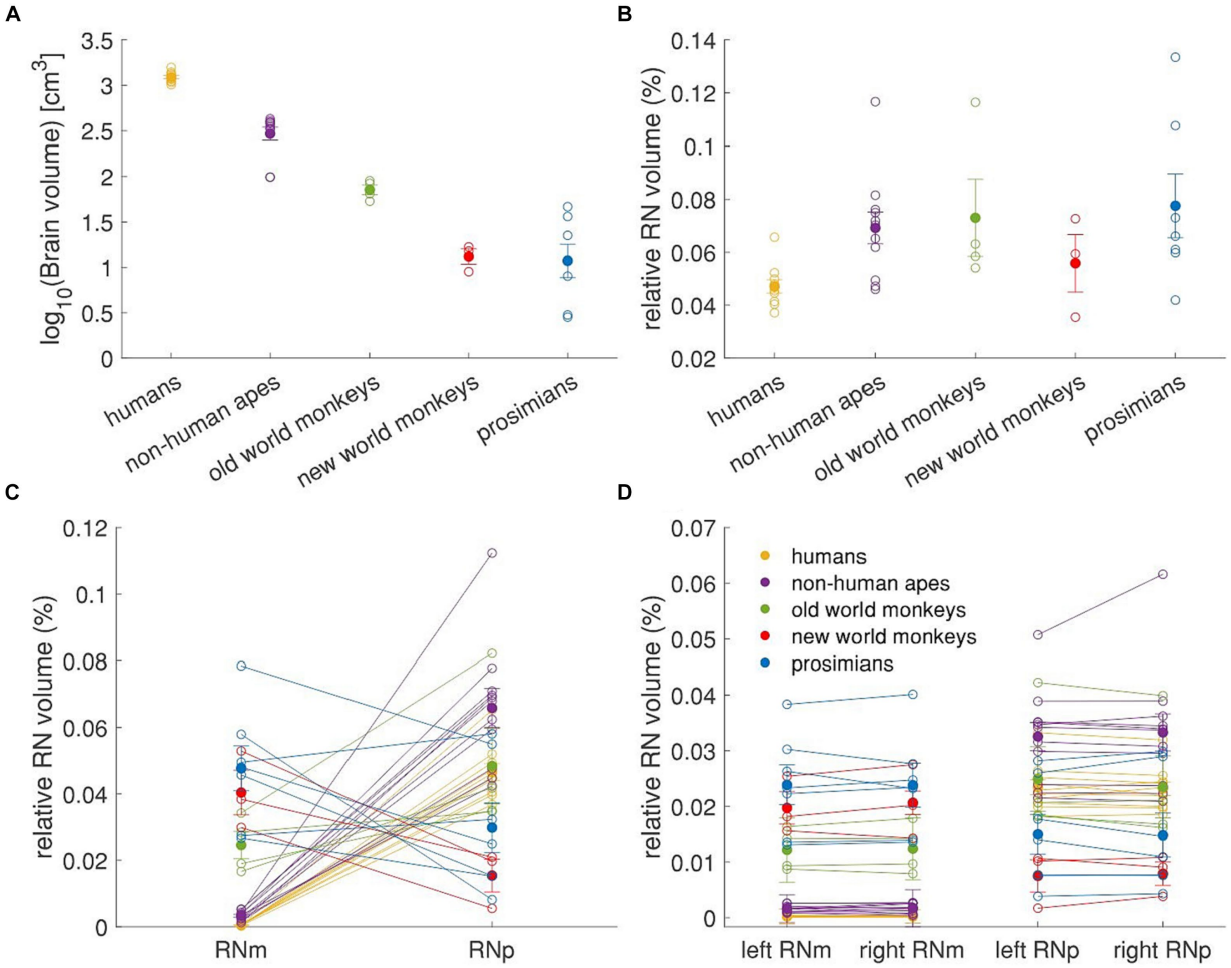
Relative volume of RN and its subdivisions. The figure shows values for brain volume and left and right RNm and RNp summarized in the Table 3, as well as the mean values for each group of primates. The brain volume in **(A)** is log transformed for better visualization and the volumes of RN are expressed as a percentage of whole brain volume for better inter-individual and between-group comparisons. **(A)** Mean brain volume of 5 different primate groups. **(B)** Relative RN volume in the 5 primate groups. **(C)** Relative volume of the RNm and RNp subdivisions of the RN. **(D)** Relative volume of the RNm and RNp subdivisions in the left and the right hemisphere. Empty circles represent individual values, filled circles represent the group means, error bars represent the SEM. In all panels, the number of subjects is as following: humans = 10; non-human apes = 11; old world monkeys = 4; new world monkeys = 3; prosimians = 7.

To further explore the relationship between the RN size and the five groups of primates, we scrutinized the RN volume relative to the whole brain volume of the species (Figure 8). In our dataset human specimens had the largest fresh brain volume followed by apes and old world monkeys (Figure 8A). The highest RN volume relative to the brain volume was observed in prosimians (0.078%± 0.012% of brain volume), old world monkeys (0.073% ± 0.015%) and non-human apes (0.069% ± 0.006%; see Figure 8B). Humans and new world monkeys showed the lowest relative RN volume (0.047% ± 0.003 and 0.056% ± 0.011%, respectively). Prosimians and new world monkeys showed the highest relative volume of RNm (0.047% ± 0.007 and 0.040% ± 0.007%, respectively) while in old world monkeys, it was about 2 times smaller (0.025% ± 0.004; Figure 8C). More importantly, the relative volume of RNm in non-human apes and humans was one and two orders of magnitude smaller than in the rest, respectively (non-human apes: 0.003% ± 0.0004%, humans: 0.0005% ± 0.00005%).

**Figure 9:**
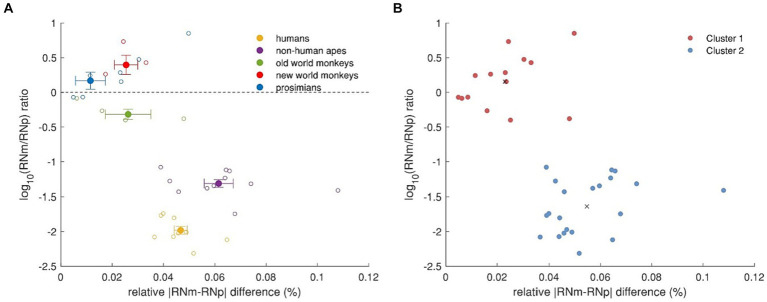
Relative volume differences between RNp and RNm across primates. **(A)** RNm/RNp ratio as a function of the relative difference between the two subdivisions. The dashed line indicates that RNp and RNm are equally large. Values above this line indicate that RNm is larger than RNp, while values under this line indicate that RNp is larger than RNm. The absolute values on the y-axis as power of 10 (10^|y|^) indicate how many times the one subdivision is larger than the other. The *x*-axis depicts the relative size of the difference between RNp and RNm. The superfamily *Hominoidea*, i.e., humans and non-human apes, show profoundly larger RNp compared to RNm and the size difference also makes higher percentage of their brain volume. **(B)** k-means cluster analysis based on **(A)**. Humans and non-human apes form a separate cluster (blue) to old world monkeys, new world monkeys and prosimians (red). Black crosses indicate the centroids of the two clusters.

It further appears that humans, non-human apes and old world monkeys had larger RNp than RNm while in contrast, new world monkeys and prosimians had larger RNm volumes than RNp. To better compare these differences across the groups, we calculated a RNm/RNp ratio and plotted it on a logarithmic scale as a function of the difference between the two subnuclei relative to the brain volume (see Figure 9A). In this way, positive values indicate that RNm is larger than RNp and vice versa, whereas 10^|y|^ indicates the magnitude of the difference. As mentioned above, old world monkeys, non-human apes and humans have larger RNp than RNm. However, while in old world monkeys the RNp was about twice as large as the RNm (the difference corresponding to about 0.026% of their brain volume) in humans and non-human apes the difference was 95-fold and twentyfold, respectively. The volume difference between RNm and RNp in humans and non-human apes was also higher compared to the rest, when considered relatively to the brain volume (0.047 and 0.061% of brain volume in humans and non-human apes, respectively). On the other hand, the RNm in new world monkeys and prosimians was 2.5 and 1.5 larger than RNp, respectively, the difference corresponding to about 0.025 and 0.012% of their respective brain volume. Thus, humans and non-human apes have the lowest relative RNm volume and the highest relative difference between the two subdivisions. We applied a k-means cluster analysis to this data to verify that humans and non-human apes together form a separate cluster compared to the rest (Figure 9B).

### Evolutionary allometry of the red nucleus in primates

**Figure 10:**
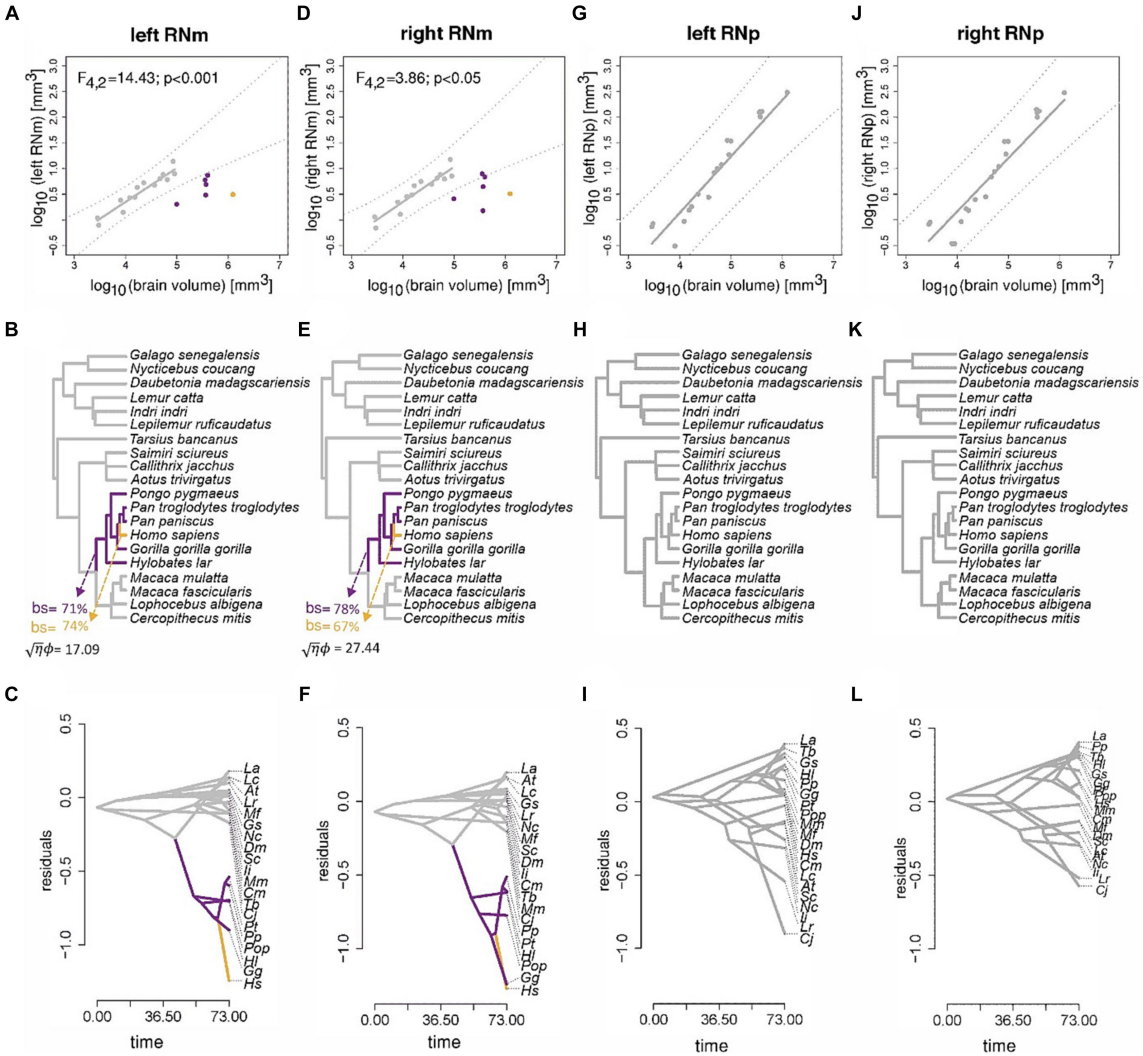
Results of the phylogenetic comparative scaling analyses of the RN. Results of the phylogenetic comparative scaling analyses of the left and right RNm [**(A–C)** and **(D–F)**], and the left and right RNp [**(G–I)** and **(J–L)**] relative to brain volume. For each region, three displays are shown in vertical order. The first depicts the bivariate plot of log10 volumes, the best-fit pGLS regression line, phylogenetic prediction intervals (following Smaers and Rohlf, 2016), and any species that indicate a significant deviation from allometry. The second display shows the results of the multi-regime OU analysis and any clades that indicate a significant shift in the mean relative volume of the RN (bs = bootstrap support). The third display shows the results of ancestral estimation of the relative volume of the RN. For each analysis (column-wise), colors indicate those species with the same mean relative RN volume. The name of species in the bottom panel have been abbreviated for better visualization purposes. Abbreviations consist of the first letters of the first and second name. *Pongo pygmaeus* was abbreviated as Pop to distinguish it from the abbreviation of *Pan paniscus* (Pp).

Next, we scrutinized the profound difference of the RNm volume in humans and non-human apes compared to the other primates. We started with an unbiased approach using the OU modeling (see Method section) to identify where allometric shifts occur based on the data (without any a-priory hypotheses). Both OU modeling and ASE estimated that RNm decreased in volume relative to brain size in the ancestral lineages of non-human apes and humans (Figures 10A–F). This result applies to both the left (Figures 10A–C) and the right RNm (Figures 10D–F). Bootstrap support for these shifts is high (>65%), and the OU modeling analysis did not reveal any other branches with strong bootstrap support. The effect size of this analysis was also high (right RNm: √η ϕ = 17.09; left RNm: √η ϕ = 27.44), confirming that the analysis has high power. The relative size of RNp, however, did not indicate any significant shifts (Figures 10G–L). This result was further confirmed by bootstrap analysis (no shift was detected with more than 3% support). This result applies to both the right and left RNp.

In the next step, we calculated the pANCOVA to test the hypotheses provided by the modeling that humans and non-human apes deviate from the allometric scaling of the RNm. Indeed, the pANCOVA analysis confirmed these evolutionary hypotheses. Assuming intercept differences in both non-human apes and humans for the RNm to brain size allometry indicates a significantly better fit than assuming a one-grade allometry (left RNm: F_4,2_ = 14.43, *p* < 0.001; right RNm: F_4,2_ = 3.86, *p* < 0.05). The human RNm revealed the greatest allometric deviation (Figures 10A,B) and was indicated to deviate significantly from the allometric prediction relative to other primates (left RNm: F_4,3_ = 23.50, *p* < 0.001; right RNm: F_4,3_ = 5.66, *p* < 0.05).

### Scaling between the cerebellum and RN

**Figure 11:**
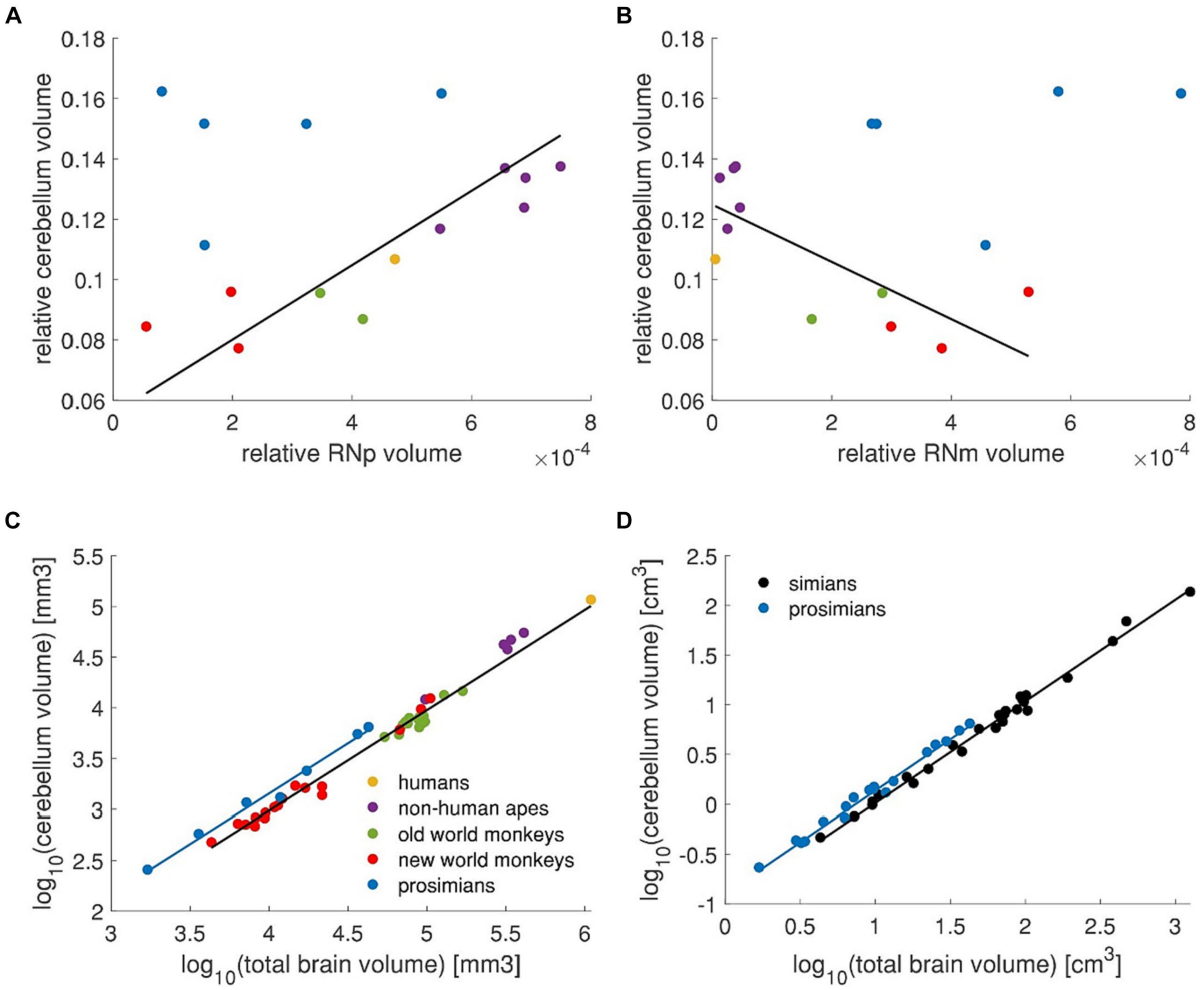
RN volume in relation to the cerebellum. **(A)** Relative cerebellum volume plotted against the relative RNp volume. High correlation (Pearson correlation, *r* = 0.91, *p* < 0.001) is observed in simians (i.e., new world monkeys, old world monkeys, non-human apes and humans – red, green, purple and yellow data points). The regression line applies to the simians (i.e., excluding the blue data points) and was fitted with pGLS. **(B)** Same data on cerebellum as in **(A)** plotted against the relative RNm volume. In this case, the two variables are inversely correlated in simians (Pearson correlation *r* = −0.75, *p* < 0.01). It should be noted however, that the RNm is consistently small in human and non-human apes such that within this group, the correlation actually might not exist (purple and yellow data points, Pearson correlation, *r* = 0.52, *p* > 0.05). The regression line was fitted vie pGLS to simians (i.e., excluding blue data points). **(C)** The cerebellum volume is plotted against the total brain volume showing different scaling in prosimians than in simians. **(D)** Cerebellum volume plotted against the total brain volume showing different scaling in simians and prosimians [data from Stephan et al. (1981)]. Regression lines were calculated with pGLS in all panels. Plots in **(A–C)** Include data from Navarrete et al. (2018), Akeret et al. (2021), and Stephan et al. (1981) as described in the text. Legend in **(C)** applies to panels **(A–C)**.

Since the RN is connected to the cerebellum and these connections underwent crucial changes during evolution (Basile et al., 2021), it is possible that voluminal variations in cerebellum across different animals could reflect these changes. Thus, we were intrigued to study whether the cross-species variation in RN volume acquired in our study is related to species-specific cerebellar volume. To this end, we took published data on cerebellar volume from Navarrete et al. (2018) that included some of the species we studied. Since these data contained only 3 prosimian species, from which only one corresponded to our RN data set, we added further four prosimian species and one non-human ape from Stephan et al. (1981). The human data were taken from Akeret et al. (2021). No cerebellum data could be matched to 2 species of old world monkeys and 2 species of prosimians in our data set. Due to different sources and methods, we normalized both the RN data and cerebellum data to the brain size (Figures 11A,B).

When the relative RNp volume is plotted against the relative volume of cerebellum (Figure 11A) it seems that these two measures are in no particular linear relation (Pearson correlation *r* = 0.25, *p* > 0.05). However, if the two primate sister groups simians (i.e., humans, non-human apes and old and new world monkeys) and prosimians are considered, it appears that a linear relationship might be present in simians (Pearson correlation *r* = 0.91, *p* < 0.001). This relationship in simians is inverse for the RNm (Figure 11B, Pearson correlation *r* = −0.75, *p* < 0.01), however note that the volume of RNm in humans and non-human apes is quite consistently small across different cerebellum volumes (Figure 11B). It therefore appears that such correlation, if present, does not apply to this subgroup (Pearson correlation *r* = 0.52, *p* > 0.05).

The question remains however, why prosimians display seemingly no relation between the RNp and the cerebellum in contrast to simians? To further scrutinize this point, we compared the cerebellum scaling with respect to the total brain size across primates (Figures 11C,D). We included all data from Navarrete et al. (2018) (i.e., *n* = 3 prosimians and *n* = 40 simians) together with the above mentioned data points from Stephan et al. (1981) (i.e., *n* = 4 prosimians and *n* = 1 simian) and human data from Akeret et al. (2021) (Figure 11C). This revealed that prosimians indeed display a different cerebellar scaling compared to simians (pANCOVA, F_4,42_ = 3.88, *p* < 0.05; Figure 11C). To ensure that this difference is not an artifact resulting from combining data from different sources, we performed the same analysis on data only from Stephan et al. (1981) that contained 18 prosimian and 27 simian species (Figure 11D). In this data set, the prosimians again showed different cerebellar scaling (pANCOVA F_4,41_ = 18.72, *p* < 0.0001).

## Discussion

During the evolution of primates, the RN seems to have been transformed from a magnocellular dominated to a parvocellular dominated RN. This change seems to involve almost exclusively the *Catarrhini* branch and was even further enhanced in the *Hominoidea* superfamily that includes humans and non-human apes. This superfamily shows the lowest relative size of RNm and the largest (relative) difference between the two subdivisions. Congruently, our statistical and modeling approach revealed that humans and non-human apes significantly deviate from the allometry in case of RNm but not RNp.

The RN vertebrate phylogeny is marked by a gradual segmentation and specialization of RNm and RNp related circuitry (Basile et al., 2021). It has been suggested that during the evolution of primates, RNp and its related circuitry became progressively larger while RNm miniaturized especially in bipedal primates (Onodera and Hicks, 1999, 2009; Hicks and Onodera, 2012; Basile et al., 2021; Olivares-Moreno et al., 2021). Our results, however, show that the RNp did not become exceptionally large in apes and humans, rather it scales concomitantly with the total brain size. It is the RNm that is exceptionally small in humans and non-human apes. The regression of RNm circuitry might therefore be associated with functional specializations that might have been enhanced due to bipedalism such as complex hand movements and fractionated body part movements (Onodera and Hicks, 1999; Hicks and Onodera, 2012; Basile et al., 2021). Indeed, the rubrospinal tract, originally crucial for locomotion in early vertebrates, seem to have become involved in arpeggio movements in rodents (Whishaw and Gorny, 1996; Whishaw et al., 1998; Morris et al., 2015) and further specialized in more complex hand movements and grasping in primates [van Kan and McCurdy, 2001; van Kan and McCurdy, 2002a,b; reviewed in Basile et al. (2021)]. However grasping and fine manipulation might be executed by direct cortico-motoneuronal projections in primates that display the use of digits for grasping and manipulation (Nakajima et al., 2000; Rathelot and Strick, 2006; Courtine et al., 2007; Lemon, 2008; Rathelot and Strick, 2009; Olivares-Moreno et al., 2021) thus perhaps reducing the involvement of RNm in these animals. The ecological advantages of fine dexterous hand movements in primates might have favored genetic modifications that enable the maintenance of cortico-spinal connections that enhanced these motor capabilities (Gu et al., 2017; Yoshida and Isa, 2018). The question however still remains, whether the reduction of RNm in humans and non-human apes represent a refinement in the specialization of RNm or a simple regression due to its redundancy as the pyramidal tract might have overtaken the control of hand movements (see also Herculano-Houzel et al., 2016). In the latter case, the human RNm could be a leftover from the prenatal stages and early ontogeny (Ulfig and Chan, 2001; Yamaguchi and Goto, 2006). It appears however implausible that neurons in RNm would be maintained without functional relevance. Moreover, complex hand precision movements are certainly supported by parallel systems (pyramidal and extrapyramidal) which may differentially contribute to specific aspects of the movements (Whishaw and Gorny, 1996; Whishaw et al., 1998; Kinoshita et al., 2012; Morris et al., 2015; Yoshida and Isa, 2018). Although the rubrospinal tract has been considered rudimentary in humans (Nathan and Smith, 1982), it has been visualized by DTI (Meola et al., 2016). It is thought to be involved in compensation for pyramidal tract injuries (Rüber et al., 2012; Jang and Kwon, 2015; Prillwitz et al., 2021), however an unequivocal evidence for this function is difficult to obtain in humans. Furthermore, the RNm, the putative origin of the rubrospinal tract, could also be identified based on its connectivity to the interposed cerebellar nucleus (Cacciola et al., 2019). Thus, despite a very small size, RNm could still perform a specific function in hand movements in humans.

Since the circuitry of both RNp and RNm involves the cerebellum, it is reasonable to assume that voluminal changes in RN and cerebellum may co-occur. Interestingly, we found a relationship between the cerebellum volume and RNp in simians. The volume of RNp in prosimians however seem unrelated to the cerebellum. These findings however provide only a limited evidence for such relations as they are based on small number of species and the data on cerebellum and RN are combined from different subjects that were assessed with different methods.

The RNp receives input from the dentate nucleus of the cerebellum (Flumerfelt et al., 1973; Asanuma et al., 1983; Novello et al., 2022) and projects to the inferior olivary nucleus (Courville and Otabe, 1974; Strominger et al., 1979, 1985; Onodera and Hicks, 2009) which in turn provides major input to the cerebellar cortex (Brodal and Brodal, 1981, 1982; Whitworth et al., 1983; Whitworth and Haines, 1986; Azizi, 2007). Interestingly, the dentate nucleus and the cerebellar hemispheres seem to have expanded during the evolution of humans and apes (Baizer, 2014; Magielse et al., 2022) concomitantly with the associated frontal (Smaers et al., 2017) as well as parietal (van Essen and Dierker, 2007; Goldring and Krubitzer, 2020; Bruner et al., 2023) cortical areas. Although a great deal of the expansion of the fronto-cerebellar system might be attributed to the evolution of non-motor higher cognitive functions (Magielse et al., 2022), it is equally reasonable to assume that these cortico-cerebellar changes also partly reflect specialized motor activities that evolved in human and non-human apes (Smaers et al., 2011, 2013). In fact, a well-developed posterior parietal cortex seem to be associated with hand use and complex manipulations (Padberg et al., 2007; Goldring and Krubitzer, 2020). It therefore appears that increase in hand dexterity appears concomitantly with the expansion of fronto-parietal cortical and cerebellar networks in the evolution of primates. On the one hand, these expanded networks could omit the RNm thereby promoting its miniaturization, on the other hand, the strong associations of RNp with the cerebellum via the dentate and the olivary nucleus could support the maintenance of a large RNp.

Taken together, a correlation between cerebellum and RNp does seem plausible and further studies should explore it in more depth. However, somewhat surprising is the fact that simians and prosimians differ in this aspect. With the aid of previously published data (Stephan et al., 1981; Navarrete et al., 2018; Akeret et al., 2021) we showed that the cerebellum scaling with the total brain volume in prosimians differs from the scaling in simians. Although this is not a sufficient explanation for the different relationship between cerebellum and the RN, it indeed indicates that the cerebellar evolution in prosimians might differ from that in simians. Previous studies that analyzed the cellular scaling rules of cerebellum in primates reported no difference across primate species (Azevedo et al., 2009; Gabi et al., 2010; Herculano-Houzel et al., 2014). This means for instance that within primates, the number of neurons in cerebellum scales proportionally to the mass of cerebellum while the density of neurons remains constant independently of the mass of cerebellum (Azevedo et al., 2009; Gabi et al., 2010). Furthermore, primates as group show a clade-specific relationship between number of neurons within the cerebellum and the number of neurons in the rest of the brain (Herculano-Houzel et al., 2014). However, it should be noted that only up to 12 primate species were investigated in these studies with only 2 prosimian species involved. A recent study (Magielse et al., 2023 – preprint) investigated cerebellar volumes in 34 primate species including 9 prosimian species (mostly *Lemuriformes*). Here it was found that the volume of cerebellum relative to the rest of the brain varies across primates with the highest ratios displayed by prosimian species but also by human and non-human apes. In contrast old and new world monkeys showed much lower values. It therefore seems possible that cerebellar scaling might differ between different groups of primates and it might be worthwhile to scrutinize the relations of cerebellum and its subdivisions to the RN across primates.

Although neuroanatomical studies of RN have been performed in several primate species incl. Rhesus monkey (*Macaca mulatta*) (King et al., 1971; Miller and Strominger, 1973; Strominger et al., 1979), lesser bushbaby (*Galago senegalensis*) (Murray and Haines, 1975), Japanese macaque (*Macaca fuscata*) (Onodera and Hicks, 2009), chimpanzee (*Pan troglodytes*) (Strominger et al., 1985), baboon (*Papio*) and gibbons (*Hylobates*) (Padel et al., 1981), as well as humans (Patt et al., 1994; Ulfig and Chan, 2001; Yamaguchi and Goto, 2006; Onodera and Hicks, 2009, 2010), the perhaps most detailed account of intrarubral organization and connectivity has been achieved in crab-eating macaque monkey (*Macaca fascicularis*) (Ralston et al., 1988; Ralston and Milroy, 1989; Jenny et al., 1991; Ralston and Milroy, 1992; Ralston, 1994a,b; Burman et al., 2000a,b). The RN is constituted by neurons with a variety of dendritic and somatic morphologies (Massion, 1967; King et al., 1971; Burman et al., 2000a,b). Nevertheless, these neurons can be grouped into three main types based on connectivity and size. The rubro-olivary and rubrospinal neurons represent the projection neurons targeting the two main outputs of RNp and RNm, respectively. The third neuron type is represented by the locally connected GABAergic interneurons that are possibly more abundant within the RNp (King et al., 1971; Ralston and Milroy, 1992; Burman et al., 2000a). The projection neurons receive cortical and cerebellar inputs that are somatotopically aligned (Jenny et al., 1991; Ralston, 1994a,b; Burman et al., 2000a,b). The cortical inputs tend to reach the distal dendritic segments and can be part of glomeruli formed between the corticorubral axons, interneurons and projection neurons, while the cerebellar afferents predominantly target proximal dendritic and somatic sites. Based on the soma size, large, medium and small neurons have been repeatedly described in the RN that perhaps roughly correspond to rubrospinal neurons, rubro-olivary neurons and interneurons, respectively (Massion, 1967; King et al., 1971; Padel et al., 1981; Patt et al., 1994; Burman et al., 2000a,b; Onodera and Hicks, 2009, 2010). In our study, we analyzed slices with somatic staining therefore neither dendritic morphology nor connectivity of the neurons could be scrutinized here. However, our observations of medium-size to large neurons in the RNp and large and giant neurons concentrated mainly in the RNm is in accordance with previous reports. Furthermore, we observed that the cell density varied across the RNp forming several putative subdivisions in many investigated species. Previously, the human RNp has been subdivided based on cell density and lamellae into oral, caudal and dorsomedial part (Pu et al., 2000; Büttner-Ennever et al., 2014). On the other hand, Onodera and Hicks proposed, what they call a rolled-sheet model of the RN (Onodera and Hicks, 2009; Hicks and Onodera, 2012). Based on their comparative work, they concluded that the NB was separated from the nucleus of Darkschewitsch in humans during the evolution and shifted toward the RN and now projects to the olivary complex via the central rather than medial tegmental tract. The human NB therefore constitutes the cell-dense dorsomedial part of the human RNp. The remaining human RNp is subdivided into ventrolateral and dorsomedial aspects that are continuous with the NB and together wrap around the SCP (Onodera and Hicks, 2009). Note that the dorsomedial subdivision of RNp by Onodera and Hicks (2009) does not correspond to the classically designed dorsomedial subdivision (Pu et al., 2000; Büttner-Ennever et al., 2014). Rather, it is the NB that corresponds to the conventional dorsomedial subdivision. Apart from this conceptional differences, it is nevertheless obvious that the RNp might comprise several cytoarchitectonic subdivisions in primates. In macaques, it has been shown that the inputs from different cortical areas end within the RNp in a topographical manner (Burman et al., 2000b) and neuronal responses revealed a somatotopic representation of body parts within the RN (Larsen and Yumiya, 1980). Thus, the RNp might be composed of functionally different sub-regions but how this functional topology relates to the cell density based parcellations is unclear. Furthermore, it has been demonstrated in rodents that the dorsolateral subdivision of the RNp projects to the facial and accessory abducens nuclei and thereby contributes to eyelid movements (Morcuende et al., 2002; Pacheco-Calderón et al., 2012; Parras et al., 2022). Similar rubro-facial projection originating from a dorsolateral portion of the rostral RNp has been demonstrated in cats (Yu et al., 1972; Pong et al., 2008; Miller and Gibson, 2009). We are not aware of a study showing such projection in primates but the territorial specificity of these projection neurons within the RNp further supports the idea that RNp might be a collection of different functional and possibly structural partitions.

The present study also provides cytoarchitectonic probability maps in stereotaxic space based on delineations in human postmortem brains and introduces a high-resolution map of the RN in the BigBrain model (Amunts et al., 2013). Our observations agree with previous findings (Hicks and Onodera, 2012) that the RN consists of a well-developed RNp and a rudimentary RNm in the adult human brain. Previous volumetric analysis of RN reported absolute ipsilateral volume of 210 mm^3^ (Colpan and Slavin, 2010) and relative volume of 0.042% (Camlidag et al., 2014). These values are slightly lower but still comparable to our values of ipsilateral absolute RN volume of 295 mm^3^ and relative bilateral RN volume of 0.047%, respectively. The differences might occur due to different methodology or volume calculations. The RNm in humans is rather small (our data: ~3 mm^3^) and is possibly constituted only by few hundred of cells that wrap around the caudal pole of RNp (Massion, 1967; Herbel, 1979; Nathan and Smith, 1982; Patt et al., 1994; Onodera and Hicks, 2010). The distance between neighboring sections (0.3 mm to 1.2 mm) that we used to compute probabilistic maps might therefore be at the limit to trace the RNm properly. Consequently, the resulting probability map of the RNm showed a rather low maximal probability and a high variability. High-resolution, gap-less series of sections in the BigBrain provide here a useful alternative to study the RNm with high accuracy.

Detailed location of the RN is important to guide neurosurgery and neuroimaging studies requiring high level of anatomical detail. However, the delineation of RN in MRI data is not always straightforward. The combination of histological and MRI atlases is therefore a useful approach to increase anatomical precision in MRI scans that have been used to segment subcortical structures, including the RN, in MRI images (Xiao et al., 2012, 2019; Paquola et al., 2021). Several atlases of the human brain stem that included the RN have been published recently based on histological sections of single brain specimen (Coulombe et al., 2021), post-mortem MRI of a single brain specimen (Adil et al., 2021; Lechanoine et al., 2021) as well as large *in vivo* MRI data set creating probabilistic maps (Pauli et al., 2018). An atlas based on the combination of histological sections showing the cyto- and chemoarchitecture and MRI has been recently introduced by Agostinelli et al. (2023). Unfortunately, the level of midbrain containing RN has not been included in the analysis. Although Pu et al. (2000) demonstrated that subdivisions of the RNp (Büttner-Ennever et al., 2014) can be visualized with a gradient echo sequence, the RN is usually considered as a single entity in both histological and MRI atlases mentioned above. Furthermore, the majority of the above citated atlases is based on single specimen and they do not provide cellular resolution nor the RNm and RNp partitions. Our cytoarchitectonic delineations provide a 3D model of the human RN with its RNp and RNm subdivisions, incorporate the interindividual variability and create probabilistic and high resolution cytoarchitectonic maps accessible in the Julich-Brain (Amunts et al., 2020) and BigBrain (Amunts et al., 2013), respectively. These maps may provide a helpful anatomical framework that can support neuroimaging studies (Paquola et al., 2021) and perhaps also assist during neurosurgeries for deep brain stimulation in closely adjacent structures such as the STN (Rodriguez-Oroz et al., 2008; Eisenstein et al., 2014; Garcia-Garcia et al., 2016; Güngör et al., 2018; Avecillas-Chasin et al., 2019; Neumann et al., 2019).

## Conclusion

To our best knowledge, this is the first comparative study of the RN that involves a large number of primate species and directly compares the volume of RNp and RNm across the main primate groups. We show that humans and non-human apes have miniaturized RNm and significantly deviate from the scaling of the RNm with the total brain volume. On the other hand, the RNp is well developed in these species but its volume in humans and non-human apes is within the expected range relative to the brain volume. The differences between the primate groups might reflect the ecological adaptations and development of motor skills, perhaps the hand dexterity. However this still might not satisfactorily explain the functional relevance of the change toward a parvocellular dominated RN. Functional studies in humans revealed activation of the RN during tactile discrimination with fingers (Liu et al., 2000), isolated vowel utterance (Sörös et al., 2006), working memory task and memory recall (Sung et al., 2022) but also during pain (Dunckley et al., 2005) and migraine attacks (Cao et al., 2002). Further MRI studies in humans demonstrated structural (Habas and Cabanis, 2006, 2007) and functional (Nioche et al., 2009; Sung et al., 2022) connectivity with widespread cortical areas including the frontal, parietal, temporal and occipital cortex and an altered functional connectivity of the RN in migraine patients (Huang et al., 2019). Taken together, the evidence suggests that the (human) RN might contribute to a variety of precise motor functions but also to other non-motor sensory and perhaps cognitive functions. Our cytoarchitectonic maps of the human RN might provide an helpful anatomical scaffold for future neuroimaging studies clarifying the functional properties of the RN. A better understanding of the RN and its relation and reorganization relative to other extrapyramidal and pyramidal descending systems as well as to the cerebellum during the evolution might also promote better understanding and cure of motor related diseases and injuries (Philippens et al., 2019).

## Data availability statement

The original contributions presented in the study are included in the article/supplementary material, further inquiries can be directed to the corresponding author. The human probabilistic maps and BigBrain delineations are available at EBRAINS https://www.ebrains.eu/.

## Ethics statement

The studies involving humans were approved by Ethical Committee of the Medical Faculty of the Heinrich-Heine-University Düsseldorf, Building 14.82.01, Moorenstr. 5, D-40225 Düsseldorf; approval number: 4863. The studies were conducted in accordance with the local legislation and institutional requirements. The participants provided their written informed consent to participate in this study. Ethical approval was not required for the study involving animals in accordance with the local legislation and institutional requirements because the research on the non-human primate brains in this study involved only the analysis of already existing brain slices obtained from the brain collection at the C. & O. Vogt Institute for Brain Research of the Heinrich Heine University in Düsseldorf.

## Author contributions

MS: Conceptualization, Data curation, Formal analysis, Investigation, Methodology, Visualization, Writing – original draft, Writing – review & editing. AH: Writing – review & editing, Methodology, Investigation, Formal analysis, Data curation, Conceptualization. AB: Writing – review & editing, Writing – original draft, Visualization, Supervision, Methodology, Investigation, Formal analysis, Data curation, Conceptualization. FI: Investigation, Methodology, Writing – review & editing. HM: Data curation, Project administration, Software, Visualization, Writing – review & editing. CS: Data curation, Software, Visualization, Writing – review & editing. JS: Conceptualization, Data curation, Formal analysis, Methodology, Software, Visualization, Writing – review & editing. KA: Conceptualization, Data curation, Funding acquisition, Project administration, Resources, Supervision, Writing – review & editing.

## Footnotes

^1^ https://ebrains.eu/

^2^ https://jubrain.fz-juelich.de/apps/cytoviewer2/cytoviewer-main.php
